# Inflammatory lesions of the orbit: a single paediatric rheumatology centre experience

**DOI:** 10.1186/1546-0096-9-S1-P216

**Published:** 2011-09-14

**Authors:** EL Boulter, D Eleftheriou, NJ Sebire, C Edelsten, PA Brogan

**Affiliations:** 1 Rheumatology Department, Great Ormond Street Hospital, London, UK; 2 Rheumatology Unit, UCL Institute of Child Health, University College London, UK; 3 Histopathology Department, Department of Paediatric Laboratory Medicine, Great Ormond Street Hospital, London, UK

## Background

Inflammatory lesions of the orbit encompass a wide range of pathological processes, including Wegener’s granulomatosis (WG), sarcoidosis and related entitities, systemic lupus erythematosus, orbital myositis, Graves’ orbitopathy, foreign body, and idiopathic orbital inflammation (also known as orbital pseudotumour), amongst others. There is a paucity of literature on this presentation as it is rare in children and diagnosis may be challenging.

## Aim

To describe the clinical, laboratory, histopathological presentations, and final diagnoses for children presenting to a tertiary paediatric rheumatology service with an inflammatory lesion of the orbit.

## Methods

This was a retrospective descriptive case series of children with an inflammatory lesion of the orbit presenting to a single paediatric rheumatology service between January 1999 and July 2010.

## Results

Ten patients, median age at referral to 11.5 yrs (range 3.1–16.2 yrs) were identified. Median duration of symptoms at referral was 9 months (0.75 – 17 months). Imaging was performed in 9/10 cases: orbital MRI (n=4), orbital CT scan (n=1), both MRI and CT scan (n=4). All 10 patients had an orbital biopsy; two patients had repeat biopsies. The final diagnoses were: WG, (n=5; ANCA positive n=4, ANCA negative n=1); idiopathic orbital inflammation (n=3); atypical mycobacterial infection (n=1); and sarcoidosis (n=1).

## Conclusion

Inflammatory mass lesion of the orbit is an unusual presentation in children. The differential diagnosis is wide and may evolve over time. Orbital biopsy and screening for systemic features is essential prior to treatment with corticosteroids or other immunosuppressants to exclude malignancy, infection, vascular lesions, autoimmune conditions or other causes of orbital inflammation that can be associated with serious systemic manifestations.

